# The evolution of phenotypes and genetic parameters under preferential mating

**DOI:** 10.1002/ece3.1130

**Published:** 2014-06-11

**Authors:** Derek A Roff, Daphne J Fairbairn

**Affiliations:** Department of Biology, University of CaliforniaRiverside, California, 92521

**Keywords:** Coevolution, genetic correlation, heritability, mate choice, preference, quantitative genetics

## Abstract

This article extends and adds more realism to Lande's analytical model for evolution under mate choice by using individual-based simulations in which females sample a finite number of males and the genetic architecture of the preference and preferred trait evolves. The simulations show that the equilibrium heritabilities of the preference and preferred trait and the genetic correlation between them (*r*_G_), depend critically on aspects of the mating system (the preference function, mode of mate choice, choosiness, and number of potential mates sampled), the presence or absence of natural selection on the preferred trait, and the initial genetic parameters. Under some parameter combinations, preferential mating increased the heritability of the preferred trait, providing a possible resolution for the lek paradox. The Kirkpatrick–Barton approximation for *r*_G_ proved to be biased downward, but the realized genetic correlations were also low, generally <0.2. Such low values of *r*_G_ indicate that coevolution of the preference and preferred trait is likely to be very slow and subject to significant stochastic variation. Lande's model accurately predicted the incidence of runaway selection in the simulations, except where preferences were relative and the preferred trait was subject to natural selection. In these cases, runaways were over- or underestimated, depending on the number of males sampled. We conclude that rapid coevolution of preferences and preferred traits is unlikely in natural populations, but that the parameter combinations most conducive to it are most likely to occur in lekking species.

## Introduction

In many organisms, one or both sexes are able to exercise choice among potential mates. Because of a genetic correlation between the preference and the preferred trait, such circumstances are expected to give rise to the joint evolution of the preference in one sex and the preferred trait in the other (Andersson 1994; Shuster and Wade 2003). The genetic correlation arises because mate choice inevitably produces assortative mating between the preference of the choosing sex (hereafter females, for simplicity, though males also frequently show preference for particular female traits (Bonduriansky 2001)) and the values of the preferred trait expressed in the chosen sex (hereafter males). For clarity, we call this pattern *preferential mating* to distinguish it from assortative mating for the same trait in the two sexes, as for example, size-assortative mating (Jiang et al. 2013). The general consensus is that the genetic correlation generated by preferential mating will arise primarily through linkage disequilibrium rather than pleiotropy (Lande 1981; Kirkpatrick 1987; Pomiankowski and Iwasa 1993; Mead and Arnold 2004; Radwan 2008; Kuijper et al. 2012). The basis for this assumption is simply that the mechanisms by which the choosy sex perceives and compares the traits displayed by the chosen sex (i.e., characteristics of the sensory and central nervous systems) typically differ greatly from the characteristics of the preferred traits (e.g., morphological structures and behaviors), and hence the two types of traits need not share genetic, developmental or physiological pathways.

In the simplest case of preferential mating there is no direct selection on females (i.e., preferential mating carries no fitness costs or benefits for females) and only weak stabilizing selection on the males. Prum (2010) termed this scenario the null model of evolution by sexual selection. The quantitative genetic version of this scenario was analyzed by Lande (1981) and a simpler two locus model by Kirkpatrick (1982). Because preferences and preferred traits typically have an underlying polygenic basis (Bakker 1999), we focus upon the quantitative genetic model of Lande (1981). An important conclusion of Lande's analysis was that a line of equilibria exists rather than a single equilibrium combination of preference and preferred trait. This line of equilibria may be stable in the sense that a population displaced from it will return to the line, though not necessarily to the same point on the line, or it may be unstable in that, if displaced, the population continues to diverge away from the line. The latter situation is Fisher's runaway selection. Lande's analysis shows that coevolution of the preference and preferred trait requires a genetic correlation between them, and that the probability of Fisher's runaway selection is determined by the relative sizes of the genetic variances in preference and the preferred trait, the strength of stabilizing natural selection on the males, and the strength of female “choosiness” (see below for details).

Lande (1981) worked out the conditions under which a genetic correlation between preference and the preferred trait will be generated but his analysis did not address the expected magnitude of that correlation. The magnitude of the genetic correlation is not relevant to the equilibrium conditions predicted by Lande's analysis, but it is important in determining the rate of coevolution of the preference and preferred trait. If the genetic correlation is very low then coevolution will proceed very slowly: thus if there is a stable line of equilibria, return to equilibrium after displacement may take many generations, and if the line of equilibria is unstable, runaway selection will proceed very slowly. Kirkpatrick and Barton (1997) estimated the expected value of the genetic correlation using the approximation, 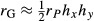, where *r*_G_ is the genetic correlation, 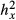 and 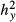 are the heritabilities of the preference and the preferred trait, respectively, and *r*_P_ is the phenotypic correlation between them. The Kirkpatrick and Barton estimate, hereafter designated *r*_KB_, sets an upper limit of 0.5 for the genetic correlation and in most cases the genetic correlation is likely to be much lower, suggesting that the coevolution of preference and preferred trait may be a very lengthy process.

A critical assumption of the null model as presented in Lande's analysis is that selection is sufficiently weak that the heritabilities of the two traits remain constant, with losses of additive genetic variance due to selection being replaced by mutation. This assumption is unlikely to be true under realistic scenarios of preferential mating because mate choice would be expected to exert significant selection on the preferred trait, potentially reducing trait heritability. In addition, as a form of assortative mating, preferential mating has the potential to either reduce or inflate the heritability of the preferred trait. Hayashi (1998) showed that assortative mating for the same trait in both sexes inflates the heritability of the trait involved. The situation is somewhat more complex in preferential mating because two traits are involved, the preference and the preferred trait, and the effect on heritability depends on the relative additive genetic (and to a lesser extent phenotypic) variances of the two traits. Specifically, if the variance of the preference is less than that of the preferred trait preferential mating would be expected to reduce the heritability of the preferred trait, whereas if the variance in the preference is greater than that of the preferred trait the heritability of the preferred trait will be increased. The reason for these effects is that when the variance in preference is less than the variance in the preferred trait, individuals with preferred traits in the tails of the distribution will be chosen in lower frequency than their representation in the population, thereby decreasing the variance and deflating the heritability. On the other hand, if the variance in preference is greater than the variance in the preferred trait, individuals in the tails of the distribution will be chosen in greater frequency relative to their representation in the population and hence the variance in the preferred trait and its heritability will be increased. These effects will be greatest when the two distributions have the same mean but will still be evident if they are displaced relative to each other, because the increase in frequency in a single tail will still flatten the distribution and increase the variance.

The degree to which the heritabilities of the preference and preferred trait and the genetic correlation between them are affected by preferential mating will be influenced by the number of males each female can potentially survey before making her choice. Lande's model represents the extreme in which each female can survey the entire male population (following natural selection on the males) and mates with a given male with a probability specified by the preference function. However, if females must chose their mates based on only a small sample of males, realized mating will more closely resemble random mating. The strength of selection through mate choice will therefore be low, the heritabilities should remain close to those expected under random mating, and little genetic correlation is likely to build up due to linkage disequilibrium.

In this article, we extend and add more realism to Lande's model, and hence to the null model of evolution by sexual selection (Prum 2010), using individual-based simulations in which females sample a finite number of males and the genetic architectures of the preference and preferred trait are allowed to evolve. We use these simulations to address the following three questions:

1. Are the equilibrium heritabilities and genetic correlation influenced by the initial model parameters?
2. Does Lande's analytical model accurately predict the quantitative genetic combinations that lead to Fisher's runaway process?
3. How well does the *r*_KB_ approximation predict the equilibrium genetic correlation?

## Stochastic Simulation Models for the Joint Evolution of Preference and the Preferred Trait

Definitions and acronyms for all model parameters are given in Table 1. Because the focal genetic parameters typically measured and reported in behavioral studies are the heritabilities and the genetic correlation, and the Kirkpatrick and Barton (1997) approximation is for the genetic correlation, we present our results in terms of heritabilities and the genetic correlation rather than the variances and covariances. Throughout our analyses, we use the subscript *x* for parameters referring to preference and *y* for those referring to the preferred trait and, for simplicity we assume that females are the choosy sex. We assume a diploid organism in which the two traits are each equal to the sum of the effects of 100 unlinked loci with no loci affecting both traits (i.e., no pleiotropy) plus an environmental effect. The genes for both traits occur in both sexes but each is phenotypically expressed only in one sex. Genetic values at each locus of the preferred trait in the starting population were drawn from a normal distribution with a mean of zero and a variance of 1. Because the variance in mating preference relative to that of the preferred trait is predicted to affect the heritability of the latter trait, we varied the genetic variance at the preference loci from 0.1 to 4.0. The per locus ratio of additive genetic variance in preference to additive genetic variance in preferred trait is the genotypic ratio, which we designate as *G*_ratio_.

**Table 1:** List of variables and acronyms

Variable	Description (values, where applicable)
KB	Kirkpatrick and Barton estimator of the genetic correlation between preference and preferred trait
SE/BM	“Sequential/best male”: mode of mate choice in which the female samples males sequentially (SE) with a finite probability of accepting each male sampled, and that her default, if no males are chosen on first pass, is to choose the best male from those already sampled
SI/BM	Simultaneous/best male”: female compares all surveyed males before making her choice, which is equivalent to simultaneous sampling (“best-of*-n*” model)
SE/LM	“Sequential/last male”: female inspects males sequentially, as in the SE/BM scenario, but if no male is accepted during this sequential inspection, she accepts the last male examined rather than the male that most closely matched her preference
AP	Absolute preference function
RP	Relative preference function
*x*	Female preference
*y*	Preferred trait in male
*w* (*y*)	Stabilizing selection function on males
*ω*	“Width” parameter in stabilizing selection function
*P* (*y*|*x*)	Probability of a female with preference *x* accepting a male with trait *y*
*ν*	Measure of tolerance or choosiness of female preference indicating the width of the female preference function. As *ν* increases (10–60), choosiness decreases.
y*	Mean value of *y* available to a given female in the RP model
*N*	Maximum number of males sampled per female (5,20,100)
*V*_g*x*_	Per locus variance in preference (1)
*V*_g*y*_	Per locus variance in preferred trait (0.1–4)
*V*_e*i*_	Environmental variance in trait *i* (=*x* or *y*)
*V*_G*i*_	Additive genetic variance in trait *i* (=*x* or *y*) (=200 *V*_gi_)
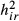	Heritability of trait *i* (=*x* or *y*) under random mating
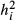	Heritability of trait *I* due to preferential mating
*G*_ratio_	*V*_G*x*_/*V*_G*y*_ = *V*_g*x*_/*V*_g*y*_
*P*_ratio_	Above ratio for phenotypic variances
*n*	Number of loci per trait (100)
*μ*	Per locus mutation rate
*V*_m_	Mutational variance (10^−5^)
*λ*	Mean number of mutations for a trait
*r*_G_	Genetic correlation between *x* and *y*
*r*_Gobs_	Genetic correlation obtained from the simulation
*r*_KB_	Genetic correlation estimated using the Kirkpatrick-Barton formula
*r*_P_	Phenotypic correlation between *x* and *y*

To distinguish the initial heritabilities from the heritabilities that evolve during a simulation we designate the initial heritabilities as 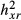 and 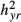 (*r* signifies that these are the heritabilities sustained under random mating) and the heritabilities resulting from preferential mating simply as 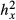 and 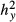. The per locus mutation rate was set as in Reeve (2000) (Appendix S1). To approximate the infinite population size in Lande's model, we used a finite population size of 10,000 individuals with an equal sex ratio at birth. This size is large enough that the effects of genetic drift could be ignored (Appendix S2).

Assumptions common to Lande's model and ours are that all genetic effects are additive, there is no pleiotropy, every female is inseminated, there is no paternal care, and the number of progeny is independent of female choice (therefore, there is no selection on female choice). As in Lande's model, stabilizing natural selection on males is determined by a Gaussian function. We considered two selection regimes, very weak and strong selection (Appendix S3). In the first case, evolution is driven entirely by female preference, while in the second evolution is driven by the combined effects of female preference and natural selection on the males.

In Lande's model, an individual female can survey the entire male population following natural selection on the males and she chooses (i.e., mates with) a given male with a probability *P* (*y*|*x*), where *y* is the male trait value and *x* is the female preference trait value. However, it seems unlikely that a female in a large population actually has access to all the males in the population. A survey of studies in which the number of males surveyed has been estimated shows that on average females sample <5 males before making their choice (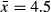, median = 2.9) and that the average range within a given species is from 1.4 to 10.1 (Table 2). The largest recorded number of males sampled was 24 (fiddler crabs, Table 2). We therefore approximated the mode of mate choice modeled by Lande but with each female surveying a finite maximum number of males (*N*), with a probability *P* (*y*|*x*) of mating with each male encountered. Each female mated with only one male and if no male was accepted after *N* males had been examined, the female chose the male with the highest probability of being accepted from within her set of *N* males. We call this mode of mate choice the “sequential/best male” (SE/BM) mode to indicate that the female samples males sequentially (SE) and that her default, if no males are chosen on first pass, is to choose the best male from those already sampled. It is important to note here that *N* is the maximum number of males a female can sample before making a choice, not the average number of males sampled before the choice is made (i.e., as in Table 1). With sequential sampling, the latter will be less than the former, as at least some females will choose a male before sampling all *N* males.

**Table 2:** Survey of species for which the number of males sampled by the female have been measured

Species	Common name	Mean	SD	Min	Max	No of females	Ref.
*Epipedobates trivittatus*	Poison-dart frog	1.3	0.6	1	3	23	1
*Branta leucopsis*	Barnacle goose	1.6	0.8	1	6	38	2
*Bufo calamita*	Natterjack toad	1.7	1.1	1	6	41	3
*Salaria pavo*	Peacock blenny	1.8	1.0	1	4	16	4
*Ficedula hypoleuca*	Pied flycatcher	2.3	1.5	1	9	125	5
*Troglodytes troglodytes*	Wren	2.3	1.2	1^1^	5	37	6
*Pomatoschistus minutus*	Sand goby	2.5	2.6	1	13	26	7
*Oophaga pumilio*	Strawberry poison frog	2.6	1.2	1	5	20	8
*Ptilonorhynchus violaceus*	Satin Bower bird	2.6	1.4	1	8	63	9
*Ips pini*	Pine engraver beetle	2.8	1.5	1	14	92	10
*Pavo cristatus*	Peacock	3.0	1.2	1	5	11	11
*Gallinago media*	Great snipe	3.0	2.4	1	10	33	12
*Centrocercus urophasianus*	Sage grouse	3.9	2.3	1	9	16	13
*Rupicola rupicola*	Cock-of-the-rock	4.4	2.3	1	12	88	14
*Tetrao tetrix*	Black grouse	4.9	2.0	2	9	31	15
*Acrocephalus arundinaceus*	Great reed warbler	5.9	2.6	3	11	11	16
*Uca pugilator*	Fiddler crab	6.7	na	1	18	14	17
*Uca annulipes*	Fiddler crab	7.5	6.0	1	24	50	18
*Amblyrhyncus cristatus*	Marine iguana	13.0	3.5	6^1^	20	12	19
*Parotia lawesii*	Lawe's parotia	17.0	na	na	na	na	20
	Mean	4.5	2.0	1.4	10.1		
	Median	2.9	1.5	1	9		

na, not available.

(1) Roithmair (1994); (2) Choudhury and Black (1993); (3) Arak (1988); (4) Fagundes et al. (2007); (5) Dale and Slagsvold (1996); (6) Benton and Evans (1998); (7) Forsgren (1997); (8) Meuche et al. (2013); (9) Uy et al. (2001); (10) Reid and Stamps (1997); (11) Petrie et al. (1991); (12) Fiske and Kalas (1995); (13) Gibson (1996); (14) Trail and Adams (1989); (15) Rintamaki et al. (1995); (16) Bensch and Hasselquist (1992); (17) Christy (1983); (18) Backwell and Passmore (1996); (19) Wikelski et al. (2001); (20) Pruett-Jones and Pruett-Jones (1990).

1 Min and Max based on ±2 SD.

For a more limited set of parameter combinations, we examined two other modes of mate choice. In the first, the female examines all *N* males prior to making her choice and then selects the male that most closely approximates her preference. We label this the “simultaneous/best male” (SI/BM) mode because the female compares all surveyed males before making her choice, which is equivalent to simultaneous sampling (it is also been called the “best-of*-n*” model; Janetos 1980). In the third mode of mate choice, the female inspects males sequentially, as in the SE/BM scenario, but if no male is accepted during this sequential inspection, she accepts the last male examined rather than the male that most closely matched her preference. This is the “sequential/last male” (SE/LM) mode of mate choice. Relative to the SE/BM mode, the SI/BM mode should increase the intensity of selection imposed by female preference because the male that most closely matches the female's preference is always chosen, whereas the SE/LM mode should have the opposite effect.

To approximate Lande's assumption that a female may survey the entire population, we set the maximum number of males sampled, *N*, at 100 and to approximate the empirical estimates we set *N* at either 5 or 20. Once each female had chosen a male, the pairs were mated and a new generation created, each female producing one male and one female offspring, which is consistent with Lande's model.

We model female preference (*P* (*y*|*x*)) as a normally distributed function, using the absolute and relative preference functions (AP and RP, respectively) described by Lande. The AP function is simpler in having one less parameter, the probability of a female preferring a male whose trait value (*y*) matches her preference (*x*) being,


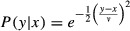
(1)

where *ν* is the width of the tolerance function for female choice. Female choosiness decreases as *ν* increases. The relative preference function is defined as


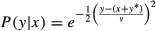
(2)

In this model, a female is most likely to accept a male whose trait value is x + y*: thus females with a positive *x* prefer males with trait values greater than y*, whereas females with a negative *x* prefer males with a trait value less than y*. Lande set y* as the mean preferred trait value of the total population of males surviving after natural selection acts. However, as noted above, because it seems highly unlikely that females are able to survey the entire population, we set y* as the mean trait value only for the *N* males she surveys. Thus, in our relative preference model, a female chooses a male based on his deviation from the mean of the males she is able to survey. To estimate y*, we initially sampled *N* males at random, estimated y* for that sample, and then allowed the female to survey the males using one of the three methods described above. Note that, with *N* = 100, our model should closely approximate Lande's relative preference model, as the mean of 100 sampled males should closely approximate the population mean.

For most simulations, the initial means of both the preference (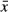) and the preferred trait (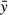) were set at the natural selection optimum for males (*θ* = 0) so that we could examine the evolution of genetic architecture due to female preference alone rather than due to the combined effect of female preference and directional selection induced by stabilizing natural selection on the males. The initial values of these parameters have no effect on the genetic architecture at equilibrium (Appendix S4).

To address our three questions, we ran two sets of simulations. For set 1, the initial values of 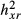 and 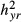 were fixed at 0.2 and 0.4, the simulations ran for 10,000 generations, and we computed mean values over the last 500 generations (a full list of parameter values and justification for these settings can be found in Appendix S5). To determine if the initial heritabilities influence the genetic correlation at equilibrium, we ran a second set of simulations (set 2) over a wider range of hertibilities, using the AP model without stabilizing natural selection and with the SE/BM mode of mate choice.

## Results

### Are the equilibrium heritabilities and genetic correlation influenced by the initial model parameters?

**Figure 1:**
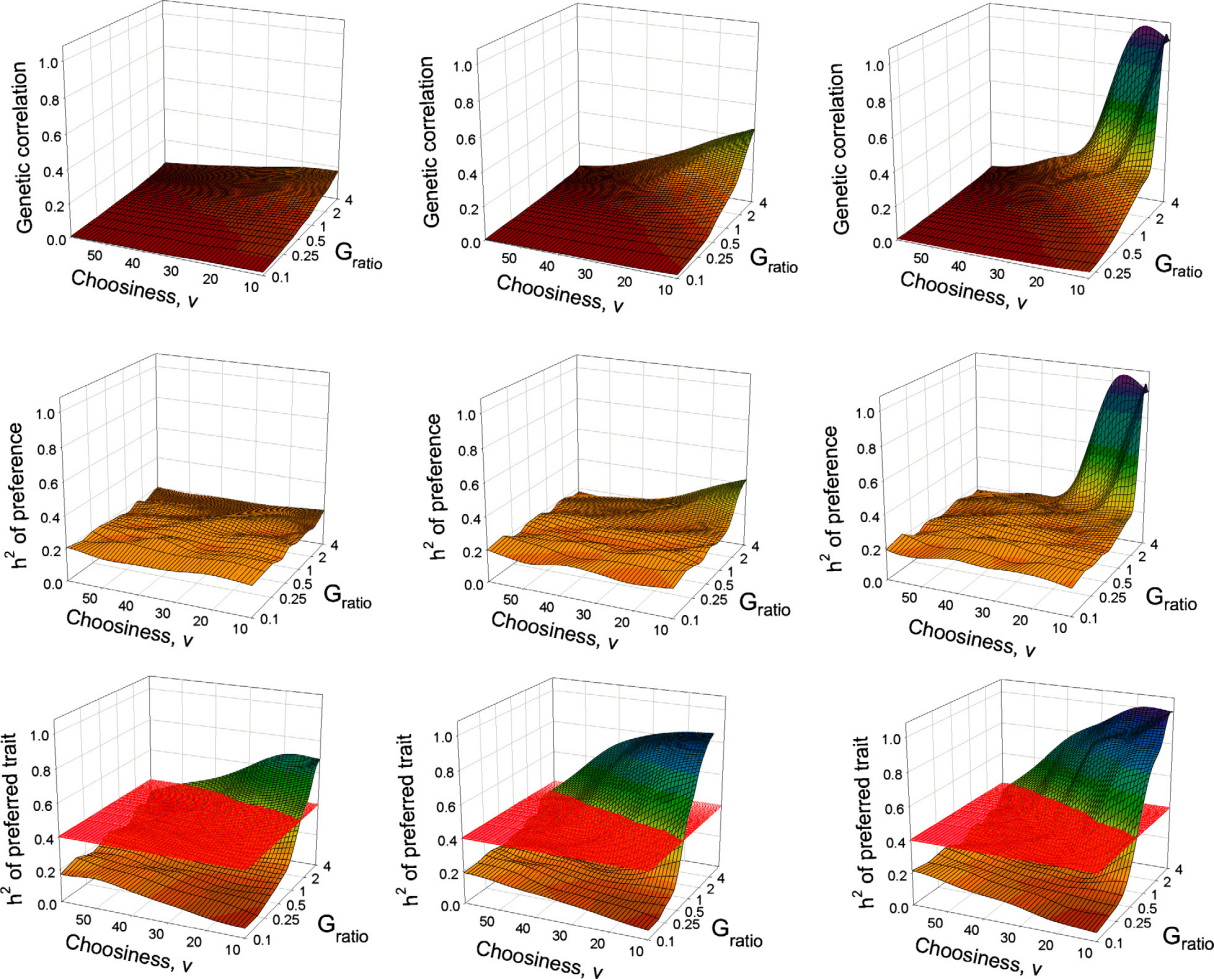
Genetic correlation and heritabilities plotted against female choosiness (*ν*), and the ratio of the genetic variance in preference to the genetic variance in the preferred trait. Note that high values of *ν* denote low choosiness (see Table 1). Results are shown for the set 1 simulations of the AP model without stabilizing selection on males. The red plane represents the heritability of the preferred trait under random mating. Females sampled a maximum of 5 (left column), 20 (middle column), or 100 males (right column).

We first consider the AP model without natural selection and with the SE/BM mode of mate choice (Fig. 1). Our simulations show that the maximum genetic correlation generated by linkage disequilibrium alone depends strongly on the number males a female is able to sample (compare panels in the top row of Fig. 1): with a maximum of five males the genetic correlation generated is ≤0.2, with a maximum of 20 males it is ≤0.4, and with 100 males it can be as high as 1. In all cases, the maximum correlations occur with high levels of choosiness (low *ν*) and high values of *G*_ratio_ (i.e., where the variance in the preference greatly exceeds that in the preferred trait). The heritabilities of the preference and preferred trait (bottom two rows in Fig. 1) also increase dramatically when the number of males sampled, choosiness and *G*_ratio_ are all high. However, through much of the parameter space, the heritability of preference (middle row) shows little change, remaining quite close to the initial value, 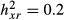, expected under random mating. In contrast, the heritability of the preferred trait is more strongly influenced by both choosiness and *G*_ratio_ (bottom row) and its behavior is complex in that it may be either depressed or elevated relative to that under random mating (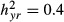). As predicted, it tends to be inflated when the genetic variance in preference exceeds that of the preferred trait (i.e., *G*_ratio_ > 1) and reduced when the reverse occurs.

In the set 1 simulations, the initial heritabilities were set at 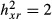 and 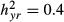 and *P*_ratio_ was twice *G*_ratio_. To determine if the initial heritabilities influence the genetic correlation at equilibrium and whether *P*_ratio_ has an effect independent of *G*_ratio,_ we examined the results from simulation set 2. Stepwise regression with the equilibrium genetic correlation as the dependent variable and the parameters 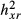, 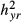, *G*_ratio_, *P*_ratio_, *ν*, *N*, and their interactions as independent variables produced a highly significant model (*F*_15,885_ = 556.9, *P* < 0.0001) that accounted for 90.4% of the variance in the equilibrium genetic correlations. Neither the heritability of preference (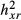), *P*_ratio_ nor their interactions was retained but all other parameters and their combinations remained in the final model. Thus, we conclude that the equilibrium genetic correlation is influenced by the initial heritability of the preferred trait but not by the initial heritability of preference. The abundance of interaction terms makes it difficult to decipher the overall effect of 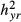. However, the main effect of 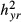 was positive both overall (*P* < 0.0001) and when separate regressions were done for each category of number of males (*P* < 0.003 in all cases), which suggests an overall positive relationship between the initial heritability for the preferred trait and the resulting equilibrium correlations. This second set of simulations also confirms that the genetic correlation increased overall with *G*_ratio_ (*r* = 0.59, df = 899, *P* < 0.0001) and indicates that the *P*_ratio_ does not have a significant independent effect.

**Figure 2:**
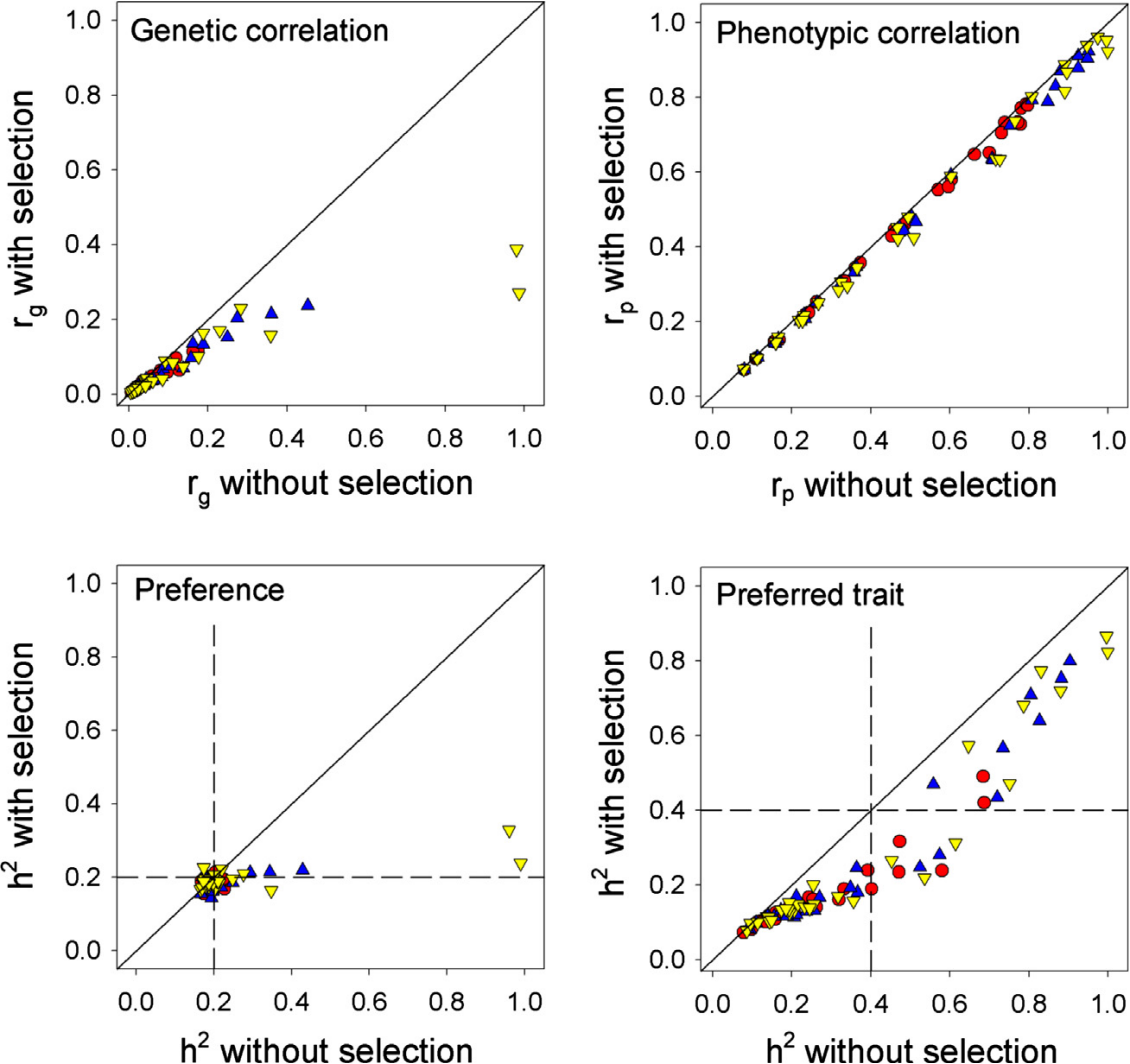
Plots of the equilibrium genetic and phenotypic correlations (top two panels) and the heritabilities of the preference and preferred trait (bottom two panels) from the AP model with selection (*y*-axis) versus the same parameters from the AP model without selection (*x*-axis). For each point, the initial parameter values were identical for the two models with the exception of the natural selection parameter, *ω*. Red circles: five males. Blue triangles: 20 males. Yellow inverted triangles: 100 males. Dotted lines show heritabilities under random mating (0.2 for preference and 0.4 for the preferred trait).

To determine if stabilizing natural selection on males affects the equilibrium correlations and heritabilites, we plotted the equilibrium parameter values with selection against those without selection for each combination of initial parameter values in the AP model (Fig. 2). Stabilizing natural selection on males reduced the genetic correlation in all 90 combinations and completely eliminated the generation of very high genetic correlations. Similarly, stabilizing selection reduced the phenotypic correlation in all combinations. The most interesting result here is that the phenotypic correlations do not show the curvilinear relationship seen in the genetic correlations, with the result that, in the presence of selection, high phenotypic correlations occur even when genetic correlations are very low.

For most parameter combinations, stabilizing natural selection on males had little influence on the heritability of preference, which tended to remain at or close to its random mating value (0.2). However, a few parameter combinations did give rise to very high heritabilities in the absence of selection, and in these cases natural selection reduced the heritabilities to close to 0.2. The heritability of the preferred trait was more uniformly reduced in the presence of natural selection, with the greatest reduction occurring at intermediate heritabilities (Fig. 2). Most noticeably, in contrast to the genetic correlation and heritability of preference, the very high heritabilities of the preferred trait were not eliminated by natural selection.

**Figure 3:**
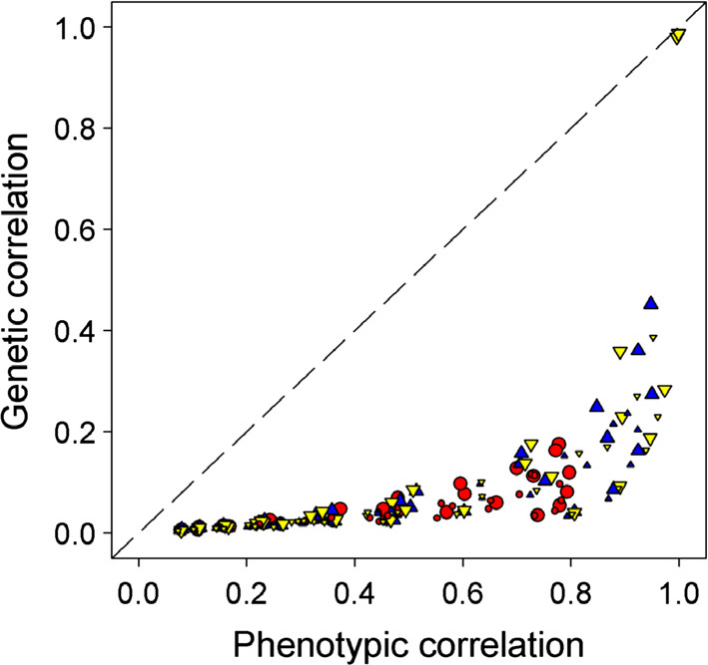
Plot of the equilibrium genetic correlation on the phenotypic correlation for the AP model without selection (large symbols) and the AP model with selection (small symbols). Red circles: five males. Blue triangles: 20 males. Yellow inverted triangles: 100 males. Dotted line shows the 1:1 relationship.

Thus, the addition of natural selection on males can have a considerable effect on the genetic parameters at equilibrium but has relatively little effect on the phenotypic correlation between the preference and preferred trait. This suggests that the phenotypic correlation is likely to be a poor indicator of the underlying genetic architecture, particularly the genetic correlation. This is illustrated in Figure 3 which shows that the phenotypic correlation is greater than the genetic correlation in all combinations and deviates considerably from the genetic correlation for almost the full range of parameter values.

**Figure 4:**
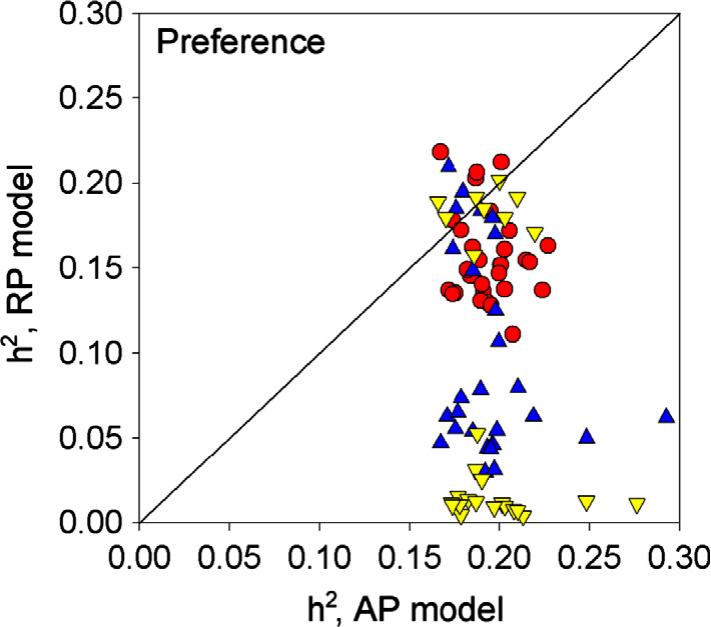
Plots of the genetic parameters and phenotypic correlation from the RP model without selection (*y*-axis) versus the same parameters from the AP model without selection (*x*-axis). Red circles: five males. Blue triangles: 20 males. 37 Yellow inverted triangles: 100 males.

The remaining model components that could influence equilibrium parameter values are the type of preference function and the mode of female choice. Our comparisons so far have been based on the absolute preference (AP) function with the SE/BM mode of mate choice. To assess the impact of type of preference function, we compared the equilibrium parameter values generated by the two preference functions in the absence of natural selection on males (Fig. 4). Analogous comparisons for the models with natural selection on males were not possible because, as explained in the next section, the RP model with selection was stable only for *N* = 5.

The two preference functions produced strikingly disparate results. In the RP model, the genetic correlation was uniformly at or close to zero. With respect to the phenotypic correlation there appears to be a break point, with correlations being the same for both models at lower values but becoming divergent thereafter (Fig. 4). With *N* = 5 or 20, the break point occurs when the phenotypic correlations reach approximately 0.2, after which the correlations in the RP model decline and then plateau at much lower values than those from the AP model. With *N* = 100 the phenotypic correlations increase in step with each other until about 0.5, after which the phenotypic correlations in the RP plateau and become consistently less than those from the AP model. In all cases, the global tendency is for the maximum phenotypic correlations generated to be much lower for RP model than for the AP model.

Preference function also impacts the equilibrium heritabilities of the preference and preferred trait (Fig. 4). The heritability of preference in the AP model varies little from its initial value (0.2), but it can be greatly diminished in the RP model, especially for *N* = 20 and 100. The heritability of the preferred trait is uniformly low in the RP model with the values that rank inversely with *N* (Fig. 4). As for the correlations, the global effect is a reduction in the heritabilities of both traits in the RP model relative to the AP model.

**Figure 5:**
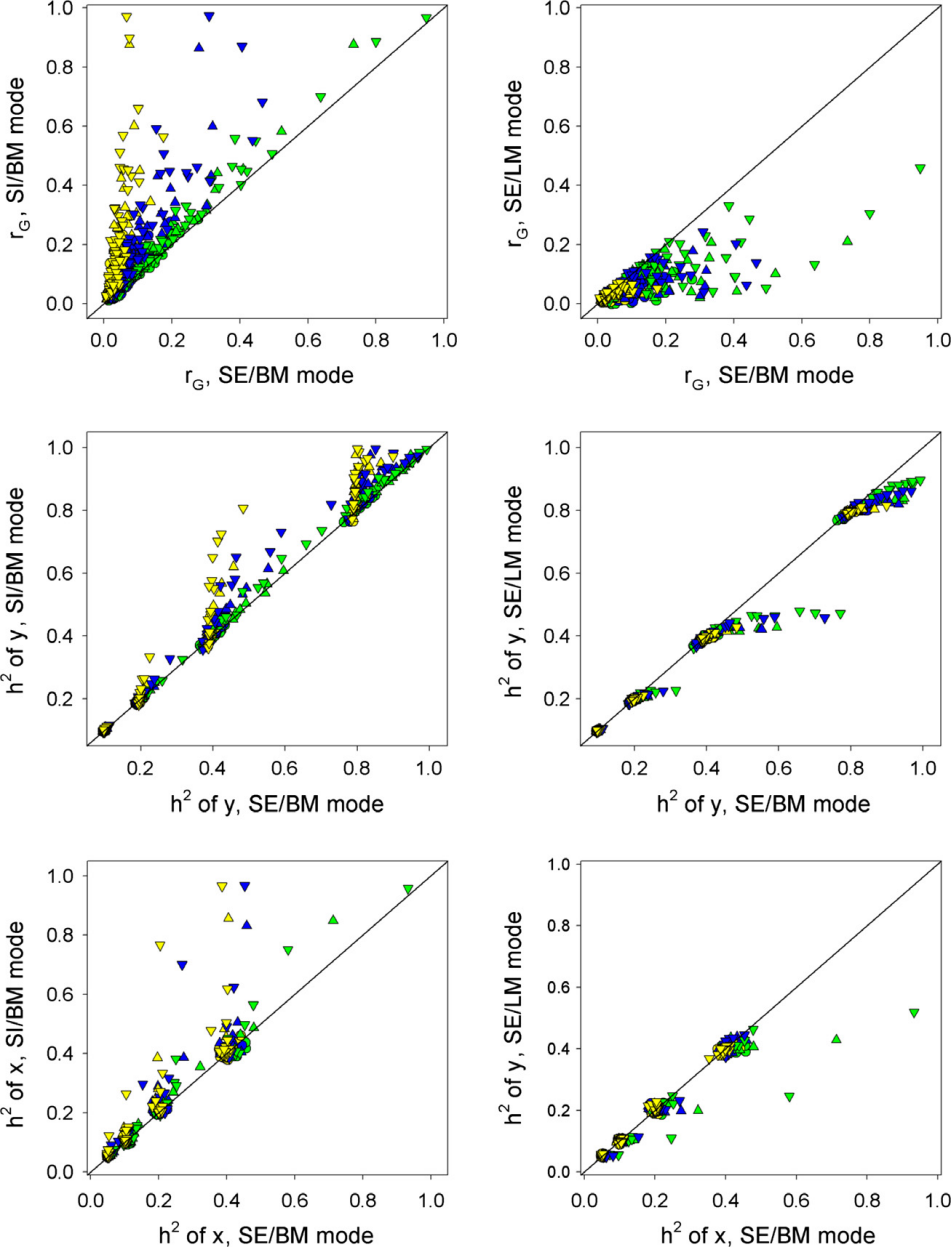
A comparison of the genetic parameters under the sequential/best male (SE/BM), simultaneous/best male (SI/BM) and sequential/last male (SE/LM) mate choice models. Solid lines show 1:1 relationship. Circles = five males, triangles = 20 males, squares = 100 males. Red, blue, yellow indicate *ν* = 10, 20, 40, respectively.

Mode of mate choice also influenced the equilibrium parameter values (Fig. 5). For the sequential best male mode (SE/BM), 95% of the combinations produced a genetic correlation <0.2, and similarly, 87% of the combinations in the simultaneous best male mode (SI/BM) and 99% in the sequential last male mode (SE/LM) yielded correlations <0.2. Thus in all three modes, few combinations led to high genetic correlations. However, relative to the SE/BM mode, the SI/BM mode increased the genetic correlation and both heritabilities, whereas the SE/LM mode decreased them (Fig. 5). There was little effect for *N* = 5 but with *N* = 20 and 100, the effect of mode of mate choice was often large, especially for the SI/BM mode. Female choosiness also affected the extent of deviation from the SE/BM mode, with a decrease in choosiness (larger *ν*) causing an increased deviation in the SI/BM mode and a decreased deviation in the SE/LM mode (Fig. 5). The reason for this difference between modes is that, when sampling sequentially, a very choosy female will be less likely than a less choosy female to select a male before the last male is sampled. When she then goes back and chooses the male that had the highest initial probability of being chosen, her choice is equivalent to the SI/BM mode. In contrast, if females who fail to choose a mate during their sequential sampling default to the last male rather than the best male, as in the SE/LM mode, increased choosiness pushes the choice toward random mating and away from the SE/BM model.

### Does Lande's analytical model accurately predict the quantitative genetic combinations that cause Fisher's runaway process?

We derived the predicted line of equilibria and the conditions for runaway selection from Lande's analysis (Appendix S6) and tested these predictions using the data from set 1. To determine if our parameter values would predict runaway selection, we substituted the fixed parameters (*ν*, *ω*) and the genetic parameter values generated in the simulations each generation (*V*_G*y*_,*V*_G*x*_, *r*_g_) into the predictive equations. We then determined if runaway selection was predicted for each generation from 100 through 10,000, scoring the result as 1.0 if yes and 0 if no. The scores were then averaged over all generations to estimate the probability of runaway selection, *P*_runaway_, for that run.

Runaway selection was not predicted for any of the simulations using the AP model (i.e., *P*_runaway_ = 0.0 in all runs). To determine if this prediction was accurate, we computed the predicted equilibrium values of 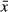 from generation 100 to the end of the simulation. We then regressed the predicted on the observed values. If a runaway process does not occur then the slope of the regression should be close to one and the intercept close to zero. As predicted, the slope averaged 0.97 (SD = 0.05) and the intercept averaged −0.02 (SD = 0.75). Lack of runaway selection was also evident from the very small changes in mean trait values as measured form the initial and final trait values: 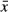 and 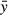 changed on average by only 0.47 (SD = 0.31) and 0.55 (SD = 0.56) standard deviation units respectively. Thus, runaway selection was neither predicted nor observed under the absolute preference model and we conclude that the analytical model accurately predicts stable equilibria under this model of preference.

For the relative preference (RP) model, the predicted outcome depended on whether or not the preferred trait was subject to natural selection. Without natural selection runaway selection was predicted for all generations in all runs (i.e., *P*_runaway_ = 1.0). The regression approach used for the AP model could not be used to test this prediction because the predicted value of 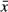 is zero when *ω* is infinitely large (Appendix S6). However, the predicted runaway process was confirmed by the relatively large changes in preference and huge changes in the preferred trait as measured by the initial and final trait values: an average of 3.16 (SD = 1.62) and 166.64 (SD = 9.24) standard deviation units, respectively. Thus, Lande's analytical model accurately predicts that runaway selection will occur under all parameter combinations when females have relative preferences and there is no natural selection on the preferred male trait.

When natural selection on the preferred trait was added to the RP model, the predictions were more mixed: stable equilibria were consistently predicted for 75.6% of the combinations (*P*_runaway_ = 0.0), runaway was consistently predicted for 5.6% of combinations (*P*_runaway_ = 1.0), and 18.8% of combinations were indeterminate in that *P*_runaway_ varied from 0.01 to 0.99. If we assume that *P*_runaway_ > 0.5 predicts runaway, 16.7% of combinations were predicted to produce runaway selection. For *N* = 5, runaway selection was predicted when the females were most choosy (i.e., *ν* = 10, its lowest value), but we did not observe runaway selection for any combination; the average change in 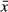 and 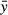 being only 0.34 (SD = 0.34) and 0.36 (SD = 0.44) standard deviation units, respectively. Thus, for *N =* 5, the runaway process was less common than predicted. In contrast, when *N =* 20 or 100 runaway was much more common than predicted. A runaway process was predicted for only 15% of combinations, again mainly when females were very choosy (*ν* = 10 [eight cases] or 20 [two cases]), but runaway was observed in all cases. The value of 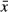 predicted differed greatly from the observed (slope = 4.39, SD = 4.92; intercept = 3.60, SD = 28.21) suggesting a runaway process. There was little change in 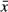 in these runs (mean change = 0.48 [SD = 0.19] standard deviation units), but 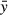 was driven an average of 6.57 (SD = 0.60) standard deviation units from its starting value. (Recall that *x* in the RP model is the female preference expressed as a deviation from the mean of the preferred trait in the sampled males [y*]. Thus, it is possible for 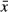 to change little [i.e., to remain as a relatively constant increment of y*] while 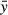 runs away.) The rapid evolution of the preferred trait led to an increasing mortality of males by stabilizing natural selection as the preferred trait moved ever further from its natural selection optimum. We terminated these runs when survival dropped below 1% on the premise that extinction was inevitable (a sample of runs to 40,000 generations showed this to be the case). Given an infinite population size as assumed in Lande's model, it is possible that an equilibrium might have been established eventually. However, with a finite population size the population was clearly headed to extinction before any equilibrium would have been achieved.

### How well does the *r*_KB_ approximation predict the genetic correlation generated by preferential mating?

**Figure 6:**
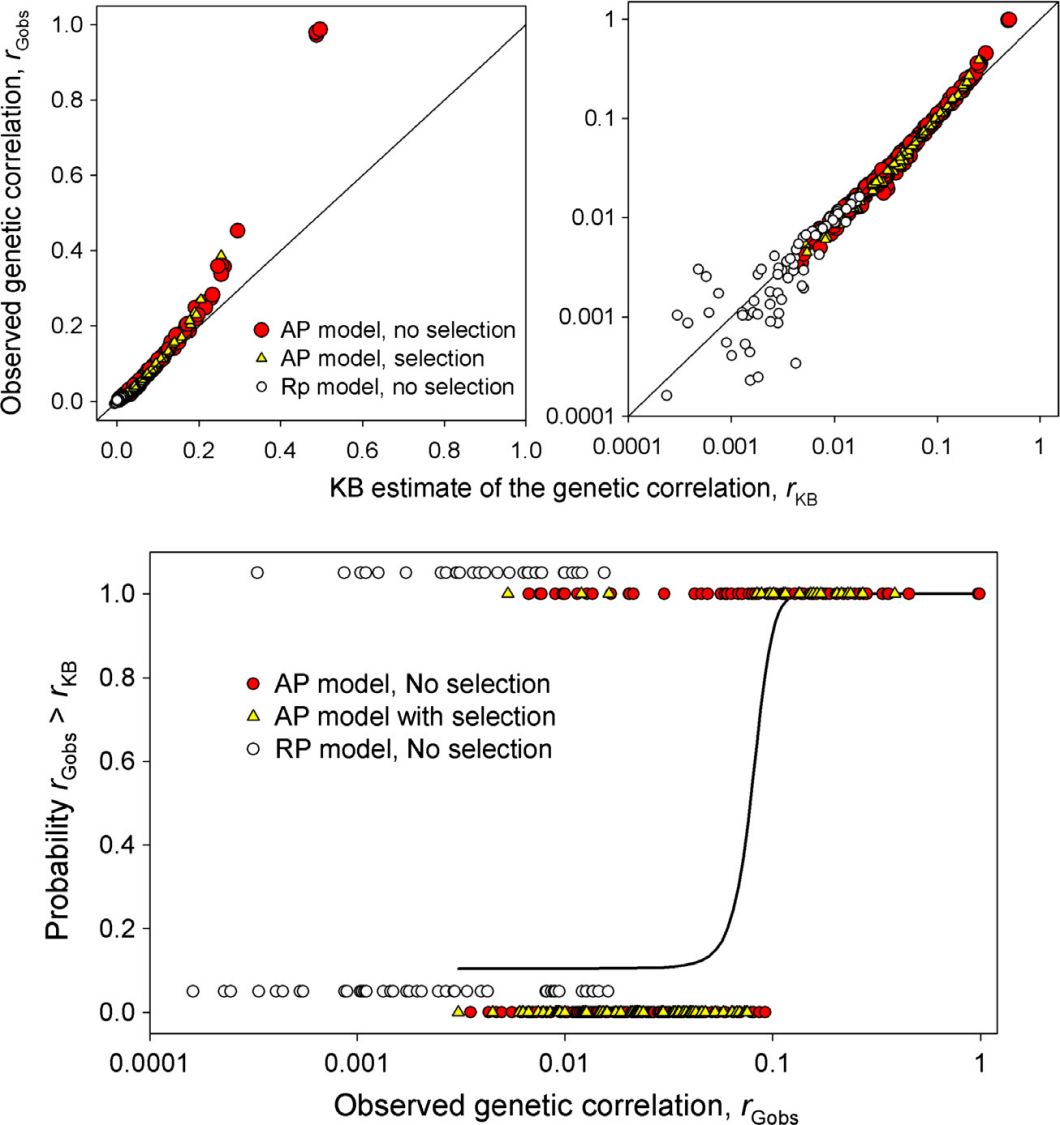
Top row: Scatter plots on linear (left) and log (right) scales showing the observed equilibrium genetic correlations, *r*_Gobs_, from our set 1 simulations plotted against the correlations specified by the KB estimator for the AP model without selection, the AP model with selection, and the RP model without selection. The solid line shows a 1:1 relationship. Bottom row: results of logistic regression analysis, the solid line giving the fitted curve for the AP model. For display purposes, the points for the RP model are shifted up slightly.

The equilibrium genetic correlations generated in the set 1 simulations ranged from zero to 0.99. To compare these with *r*_KB_, we substituted the observed equilibrium phenotypic correlations and heritabilities into the *r*_KB_ equation. The resulting estimates approximate the observed genetic correlations (*r*_Gobs_) for low values but the two progressively deviate, with *r*_Gobs_ eventually exceeding *r*_KB_ (Fig. 6). For further analysis, we used logistic regression with the probability that *r*_Gobs_ exceeded *r*_KB_ (calculated as a binary variable, with 0 designating *r*_Gobs_ − *r*_KB_ < 0 and 1 designating *r*_Gobs_ − *r*_KB_ > 0) as the response variable and *r*_Gobs_ as the predictor variable. Initially we included presence or absence of natural selection as a covariate, but the effect of selection was not significant (*F*_284,286_ = 2.404, *P* = 0.092) and so it was dropped from the model. The model with only *r*_Gobs_ as a predictor variable was highly significant (*F*_287,286_ = 163, *P* < 0.0001) and indicates that *r*_KB_ underestimates the genetic correlation from the AP model when *r*_Gobs_ exceeds 0.1 (Fig. 6). The disparity between *r*_Gobs_ and *r*_KB_ increases as the magnitude of *r*_Gobs_ increases such that for the highest values the observed values of *r*_Gobs_ were double those of *r*_KB_ (Fig. 6). Conversely, *r*_KB_ overestimates *r*_Gobs_ for *r*_Gobs_ less than approximately 0.05, though this discrepancy has little significance, given such low values of the genetic correlation.

We next tested if the type of preference function (AP or RP) was a significant predictor in addition to *r*_Gobs_. To avoid the confounding influence of natural selection, which is present only for the AP model, we compared the AP and RP models without selection. Logistic regression with *r*_Gobs_ and preference function as a covariate was highly significant and a significantly better fit than using *r*_Gobs_ alone (*F*_374,376_ = 19.19, *P* < 0.0001), indicating that the type of preference function does influence the probability that *r*_Gobs_ will exceed *r*_KB_. (Fig. 6). When the data for the AP and RP models were analyzed separately, the logistic model remained highly significant and positive for the AP model (*F*_197,196_ = 119.06, *P* < 0.0001), but was slightly negative and not significant for the RP model (*F*_89,88_ = 3.81, *P* = 0.05414). Thus, for the RP model, the probability that the *r*_KB_ will underestimate the observed genetic correlation is independent of, or only weakly related to, *r*_Gobs_, while for the AP model this relationship is strong and positive.

**Figure 7:**
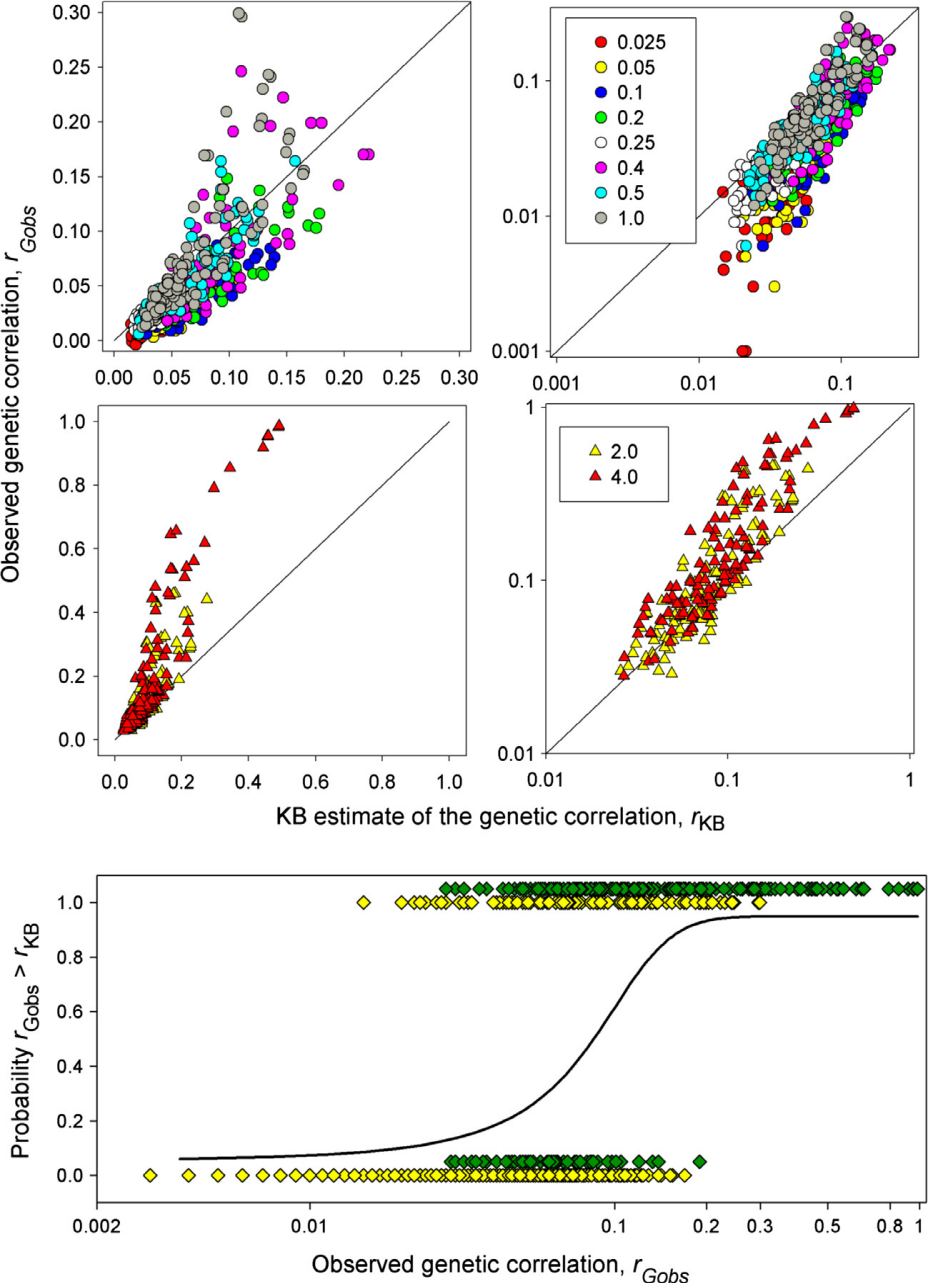
Top and middle rows: Plots of *r*_Gobs_ on *r*_KB_ for the set 2 simulations. Different colors denote different values of *G*_ratio_. Solid line shows 1:1 relationship. The right-hand column shows plots using a log scale to reduce the change in variance. Bottom row: results of logistic regression analysis, Green diamonds = *G*_ratio_ ≤ 1, yellow diamonds = *G*_ratio_ > 1 (for display purposes these points have been vertically displaced).

In the set 2 simulations, which were based on the AP model, *r*_Gobs_ was again considerably underestimated at higher genetic correlations and overestimated at low genetic correlations (Fig. 7). Because of the larger number of data points we were able to also consider possible effects of *N*, *ν* and *G*_ratio_. To this end, we ran a stepwise linear regression of log (*r*_Gobs_) on log (*r*_KB_), with *N*, *ν*, *G*_ratio_, and their interactions as possible covariates. The final model was highly significant and included all these variables plus several interactions (*F*_15,885_ = 540.3, *P* < 0.0001, *R*^2^ = 0.90). Stepwise logistic regression retained *r*_Gobs,_ *ν*, *G*_ratio_, and a number of interaction terms but not *N*. The effect of *G*_ratio_ is evident from the top four panels in Figure 7. In addition, the logistic regression of the probability that *r*_Gobs_ exceeded *r*_KB_ on *r*_Gobs_ is highly significant (*F*_900,899_ = 448.20, *P* < 0.00001) and indicates a switch from overestimation to underestimation of *r*_Gobs_ at around 0.1 predominantly due to the higher values of *G*_ratio_ (Fig 7). Overall, if the *G*_ratio_ was greater than one (i.e., the genetic variance in the preference exceeded that in the preferred trait) the genetic correlation evolved to more extreme values in our simulations than predicted by the *r*_KB_ estimator, but if the *G*_ratio_ was <1, the theoretical prediction tended to overestimate the genetic correlation at equilibrium.

## Discussion

### Are the equilibrium heritabilties and genetic correlation influenced by the initial model parameters?

Our simulations reveal that the genetic correlation between the preference and preferred trait is sensitive to most, but not all, of the model components. Although robust to changes in the initial ratio of phenotypic variances and the heritability of preference, the equilibrium correlation was strongly affected by all aspects of the mating system (female choosiness, maximum number of males surveyed, preference function, and mode of mate choice), by the presence or absence of natural selection on the preferred trait, and by several components of the initial genetic architecture (the ratio of genetic variances and heritability of the preferred trait). It tended to increase with the initial heritability of the preferred trait (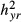), the ratio of the genetic variances (*G*_ratio_), and the maximum number of males sampled (*N*), with maximum correlations evolving when males were not subject to natural selection (i.e., the preferred trait carried no fitness cost), and females were very choosy (*ν* is low), had absolute rather than relative preferences, and were able to compare all of their potential mates before making their choice (the SI/BM mode of mate choice).

The maximum equilibrium genetic correlations were generally <0.20 and were much smaller than this over most of the parameter space. However, when *G*_ratio_ and *N* were high and *ν* was low (i.e., females were choosy and could sample many potential mates), the simulations produced much higher correlations. In the extreme, if individual females are very choosy (*ν* *<* 20), the genetic variance of the preference greatly exceeds that of the preferred trait (*G*_ratio_ > 2), and females are each able to sample 100 males, the genetic correlation between preference and preferred trait can approach unity. However, this combination of variables is highly unlikely to occur. Much more modest genetic correlations prevail if females sample 20 or fewer males. The highest correlations were generated by the SI/BM mode of mate choice with the absolute preference function and no natural selection on males, but the vast majority (>87%) of combinations in those runs still produced genetic correlations <0.2.

The equilibrium heritabilities for the preference and the preferred trait were also sensitive to many of the model components, both being affected by aspects of the mating system (maximum number of males sampled, female choosiness, preference function and mode of mate choice), and the initial genetic architecture (*G*_ratio_)*,* and by the presence or absence of natural selection on the preferred trait. As we predicted, the heritabilities of both the preferred trait and the preference could be inflated by preferential mating, the effect of preferential mating depending upon both *G*_ratio_ and female choosiness. The latter parameter is important as it governs the width of the preference function and hence the extent to which females favor males in the tail of the distribution of the preferred trait. If the additive genetic variance in preference exceeds the additive genetic variance in the preferred trait (i.e., large *G*_ratio_) and females are very choosy (i.e., small *ν*) then females at the tails of the preference distribution will choose males that lie at the extremes of the distribution of the preferred trait, thereby increasing the additive genetic variance in the preferred trait and its heritability. While both heritabilities increase dramatically if females are very choosy and the genetic variance for the preference greatly exceeds that for the preferred trait, the heritability of the preferred trait is much more sensitive to these parameters than the heritability of preference. Whereas the latter increases only at very high values of *G*_ratio_ and low values of *ν*, the heritability of the preferred trait varies over the full range of parameter values and is particularly sensitive to *G*_ratio_. If the initial genetic variance for preference exceeds that for the preferred trait (*G*_ratio_ > 1), preferential mating tends to increase the heritability of the preferred trait, but if *G*_ratio_ < 1, the heritability is reduced. These results lead to the conjecture that the effects of preferential mating on genetic architecture may provide a partial resolution for the lek paradox. Specifically, in populations where *G*_ratio_ > 1, females are very choosy, and females are able to sample 20 or more males, preferential mating could maintain high heritabilities for preferred traits in spite of strong sexual selection on those traits.

The type of preference function (i.e., whether preference is modeled as absolute or relative) also plays a highly significant role in the evolution of the heritabilities and the genetic correlation. In the absence of stabilizing natural selection on the males, the genetic correlation under the RP model never increases beyond 0.02, whereas under the AP model it can approach unity. Similarly, the heritability of the preferred trait rarely rises above 0.1 under the RP model whereas under the AP model it spans the range from 0.1 to 1. The heritability of preference under the AP model remains close to its initial value but under the RP model it varies from this value down to zero. These results call into question the conclusion of Nichols and Butlin (1989) that the lack of response to sexual selection they observed was caused only by the effects of drift in their small simulated population (*N* = 200). Their analysis was based on the RP model and, as shown here, the equilibrium heritabilities and genetic correlations under this model may be very low even in a very large population. Thus, the effects of the preference function and population size on response to selection are confounded, since both will tend to decouple the preference and the preferred trait and reduce response to selection.

If the preferred trait is under the stabilizing selection and females are able to sample large numbers of males before making their choice (*N* = 20 or 100), the RP model is unstable because the mean of the preferred trait is continually pushed farther from its natural selection optimum. In our simulations, this causes increasing mortality of males until none survive to mate with the females and the population goes extinct. This result likely reflects a deficiency in the classic model rather than biological reality. The model includes no cost to female choice in terms of inability to find a suitable male or the energetic, time and survival costs of mate searching. We would expect that in natural populations such costs (essentially selection on the female choice function) would increase as preferred males become increasingly rare, eventually stopping the runaway process and resulting in an equilibrium genetic architecture.

Under the AP model, the populations did persist even under strong stabilizing selection, and the genetic parameters were able to reach equilibrium. However, strong stabilizing selection on the preferred trait generally decreased the equilibrium genetic correlation, and the peak correlations seen at low *ν,* high *G*_ratio_, and high *N* were lost. Even at these extreme parameter combinations, genetic correlations did not exceed 0.4 if the preferred trait was subject to strong, stabilizing natural selection and it was generally reduced by a factor of about 0.6 relative to no natural selection on males. Not surprisingly, strong natural selection also reduced the heritability of the preferred trait. Over much of the parameter space, there was little effect on the heritability of the preference but, as with the genetic correlation, the peak seen at low *ν,* high *G*_ratio_, and high *N* was lost in the presence of strong selection. Thus, stabilizing natural selection on males had the overall effects of reducing the equilibrium heritabilites and genetic correlation and completely preventing the evolution of the highest genetic correlations and heritabilities for preference seen in the absence of selection.

### Does Lande's analytical model accurately predict the quantitative genetic combinations that cause Fisher's runaway process?

The line of stable equilibria generated under the AP model was well predicted by Lande's model (Appendix S6), and the condition for stability (Appendix S6) was also satisfied in all runs, both with and without stabilizing natural selection on the males. Also as predicted by Lande's model (Appendix S6), we found that runaway selection always occurred for the RP model in the absence of natural selection on the preferred trait. When natural selection on the preferred trait was added to the RP model, the analytical model predicted runaway selection in only a minority of cases (16.7%) and only when females were very choosy (*ν* = 10 or 20). These predictions were not confirmed by our simulation results. When females were able to sample only a small number of potential mates *(N* = 5), Lande's model overestimated the incidence of runaway selection, as we found stable equilibria for all parameter combinations. In contrast, the model greatly underestimated the incidence of runaway selection for *N* = 20 and 100. Runaway was never predicted for these parameter combinations but occurred in all of our simulations. This had the unfortunate consequence of causing rapid extinction of the populations because of high male mortality. Population extinction cannot occur in an infinite population, as assumed by Lande's model, but our results indicate that it would be a risk in finite populations.

In summary, our simulations confirm Lande's predictions that runaway selection should not occur if females have absolute preferences, regardless of the strength of natural selection on the preferred trait, but is expected if female preferences are relative and the preferred trait is not subject to natural selection. However, the model fails when preferences are relative and the preferred trait is subject to natural selection. Under those parameter combinations, the incidence of runaway is overestimated if females can survey relative few males before making their choice (*N* = 5), but greatly underestimated if females can sample 20 or more males. In the latter case, the runaway process is likely to be of short duration and associated with rapid population decline because males suffer increasing mortality as the preferred trait moves ever further from its natural selection optimum.

### How well does the *r*_KB_ approximation predict the genetic correlation generated by preferential mating?

The KB estimator deviated markedly from the genetic correlations generated by our simulations and, in particular, tended to underestimate the equilibrium genetic correlation when *G*_ratio_ was greater than one (i.e., when the genetic variance in preference exceeds the genetic variance in the preferred trait). In addition, whereas the maximum value that can be taken by *r*_KB_ is 0.5, our realized genetic correlations greatly exceeded this maximum when the *G*_ratio_ was >4 and females were both very choosy and able to sample 100 males. These parameter values are probably unrealistic, but nevertheless the simulations reveal the potential for evolution of much higher genetic correlations than previously thought.

Our simulations also reveal that, contrary to expectations, the accuracy of the KB estimator does depend on the female preference function. In their 1997 paper, Kirpatrick and Barton state that the approximation 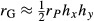 “holds regardless of how females choose their mates” (p. 1284). Our expectation would therefore be that the relationship between the KB estimator and our observed genetic correlation should be independent of the preference function. In other words, the KB estimator should be equally accurate for the AP and RP models. Contrary to this expectation, the preference model significantly affected the probability that the KB estimator would underestimate the observed correlation, underestimation being much more likely when high genetic correlations evolved under the absolute preference model.

### Can the predictions of the simulation models be tested in natural populations?

A major impediment to testing the predictions from Lande's original analytical mode and from our simulation models is the difficulty of estimating the model parameters using data from natural populations. In principle, all of the variables used in Lande's original analytical models and in our simulation models are measurable. However, surprisingly few estimates are available and to our knowledge no single study has attempted to measure all of the key parameters. The female preference function is a critical component of the co-evolution of preference and preferred trait, but as noted by Hosken and House (2011, p. R65), “There is a current paucity of information on female preference functions, their shape, whether there is genetic variation for them, and the costs of expressing different preferences” (see also Jennions and Petrie 1997; Wagner 1998). In accordance with the analysis by Lande (1981), we assumed that the individual preference function was Gaussian and the trait *x* was normally distributed in the population. These are difficult assumptions to validate in real organisms. It is important to distinguish between the individual preference functions (Eqns (1) and (2)) and the population preference function, which refers to the distribution of individual preference functions. The population preference function cannot be used to infer individual preference functions. To illustrate this, consider the example of Jackson's widowbird, *Euplectes jacksoni*, which shows a positive correlation between mating success and tail length at the population level (Andersson 1989). This population distribution is consistent with both monotonic individual preference functions and bell-shaped individual preference functions that show a skewed distribution in the population. Without directly measuring individual preference functions, these two alternatives cannot be distinguished. To obtain the individual preference functions one must repeatedly assay preference for each individual, preferably using a range of the supposed preferred trait (for a general review of methodologies see Wagner 1998).

Bell-shaped individual preference functions have been documented in a number of species. Examples include the female preference curves for song components in the cricket, *Gryllus texensis* (Gray and Cade 1999), the bushcricket *Ephippiger epjippiger* (Ritchie 2000) and the planthopper, *Enchenopa binotata* (Fowler-Finn and Rodríguez 2013; Rodriguez et al. 2013). Experiments using the cricket *Gryllus integer* suggested that the individual preference functions for a particular song component, bout length, are monotonic (Hedrick 1986; Leonard and Hedrick 2009). However, these analyses were based on binomial trials in which females were presented with a choice between a long and a short bout song. Such trials may miss individual bell-shaped preference functions. Further research has shown that female *G. integer* differ in their preference for other components of the song and that bell-shaped individual preference functions exist for these components (Hedrick and Weber 1998). Complicating this situation further is the finding that female preference is context dependent (Hedrick and Dill 1993). Context-dependent preference appears to be quite common (Gibson and Langen 1996; Jennions and Petrie 1997; Candolin 2003; Narraway et al. 2010; Farrell et al. 2011) and could play a significant role in reducing the overall genetic correlation between preference and the preferred trait.

Our simulations suggest that female preference as modeled in the RP model is unlikely unless stabilizing natural selection on the preferred trait is extremely weak and/or the number of males surveyed is low. If the female preference is relative, strong natural selection on males progressively reduces the availability of mates for choosy females and causes the populations to collapse. Even with no natural selection on males, relative preference is unlikely to generate a detectable genetic correlation between the preference and the preferred trait and the heritability of the preferred trait is likely to be driven to zero. If the number of males sampled by each female is high, the heritability of preference may also disappear. In contrast, absolute mating preferences can generate genetic correlations between the preference and preferred trait and can maintain or even increase the heritabilities of both traits, whether or not males are subject to stabilizing natural selection on the preferred trait. The considerable disparity in behavior of the AP and RP models again points to the importance of determining female preference functions in some detail.

Our simulations demonstrated that the magnitude of the genetic correlation that can be generated by preferential mating generally increases with the number of males sampled by each female. Empirical estimates (Table 1) indicated that the most realistic choice of *N* in our simulations was *N* = 5, with *N* = 20 possibly applicable in some mating systems. Our highest number of males sampled, *N* = 100, while yielding the highest genetic correlation and heritabilities, likely has little relevance in most natural mating systems. On the basis of this, we conclude that where preferential mating occurs, the genetic correlation due to linkage disequilibrium alone is highly unlikely to be >0.2 and is probably <0.1 in most cases. Unfortunately, such low genetic correlations are likely to be very difficult to detect given the sampling error inherent in empirical studies. We have been able to locate only four estimates in which preference and the preferred trait were measured on the same scale. Two of these are for the same species, the collared flycatcher, and vary greatly from 0.02 (±0.17 SE; Qvarnstrom et al. 2006) to 0.29 (±0.32; Hegyi et al. 2010). The other two estimates are from the wax moth (0.16 ± 0.07; Zhou et al. 2011) and the Texas ground cricket (0.51 ± 0.17; Gray and Cade 1999). The confidence intervals in these estimates overlap the parameter region we predict as most likely (0.0 < *r* < 0.2), but they also include much higher values and even negative values in the case of the collared flycatcher. Unfortunately, such imprecise estimates are not informative with respect to testing out our prediction that correlations >0.2 should be rare.

As a prelude to our simulations, we advanced the hypothesis that preferential mating will increase the heritability of the preferred trait if the initial genetic variance of the preference exceeds that of the preferred trait. Under this hypothesis, preferential mating may at least partially explain the unexpectedly high heritabilities often observed for preferred traits in natural populations. The simulations support this hypothesis in showing that if the additive genetic variance of the preference trait exceeds that of the preferred trait *(G*_ratio_ *>* 1) and females are choosy (i.e., *ν* is small), the heritability of the preferred trait will be inflated relative to that maintained under random mating. Our results also indicate that the critical ratio is that between the additive genetic variances rather than the phenotypic variances. Unhappily, whereas we have estimates for the phenotypic variances ratios in four species (1.0–1.1 for the collared flycatcher (Qvarnstrom et al. 2006; Husby et al. 2012); 1.63 [range 0.8–3.2] in the waxmoth (Zhou et al. 2011); 1.2 in the Texas ground cricket (Gray and Cade 1999); 0.8 in the butterly, *Colias eurytheme* (Sappington and Taylor 1990)), we have only a single estimate for the additive genetic variances. This estimate is for the collared flycatcher and is *G*_ratio_ = 0.11, which our simulations suggest would generate a very low genetic correlation. This is consistent with the estimated genetic correlation between mate choice and the preferred trait of −0.015 ± 0.169 in this species (Qvarnstrom et al. 2006). However, there are some serious statistical concerns with the method used to estimate the additive genetic variance of preference in this study (Postma et al. 2006). The female preference for male forehead patch size was taken to be the patch size of her observed mate. This assumes that females sample enough males for the accepted male's value to be a reasonable estimate of the female's true preference. If the female samples few males then what she accepts will be a poor estimate of her preferred value. Females of a related species, the pied flycatcher, sample an average of only 3.8 males (Table 2) and hence it is highly likely that the male selected is only a rough match to her true preference. If this rate of sampling also applies to the collared flycatcher then the additive genetic variance for preference will be underestimated, as will both the heritability for preference and the *G*_ratio_. Given these problems, it is not possible to truly evaluate whether or not the results of this study are consistent with our model predictions.

## Conclusions

Our review of the available empirical estimates has revealed the difficulties inherent in testing the null model of evolution by sexual selection (Prum 2010) as formulated by Lande (1981) and simulated here. Nevertheless, our results do allow some predictions that may help to guide future research. For example, our simulations suggest that under realistic scenarios of mate choice, the genetic correlation between the preference and the preferred trait resulting from linkage disequilibrium is likely to be very low. Thus, even if female preference exerts strong sexual selection on the preferred trait, the rate of change of the preference resulting from a correlated response to this selection will be low. Since this correlated response is the basis of coevolution of the preference and preferred trait, such coevolution is likely to proceed very slowly, if at all, in most natural populations. High genetic correlations can evolve in mating systems where females are very choosy and are also able to sample large numbers of males, but only if the genetic variance for preference greatly exceeds that for the preferred trait. Whether the latter criterion is likely to hold in natural populations remains to be determined. However, the former two criteria are most likely to be simultaneously true in lek mating systems where females can survey many potential mates. Lek mating systems also most closely approximate the SI/BM mode of mate choice which produced the highest genetic correlations. Therefore, we predict that rapid coevolution of the preference and preferred trait is most likely to occur in lekking species. By the same reasoning, the positive effect of preferential mating on the heritability of preferred traits, a possible resolution of the lek paradox, is also likely to be strongest in lekking species.

## Supporting Information

**Table S1.** Details on the parameter combinations used.

**Appendix S1.** Mutation rate.

**Appendix S2.** Population size and drift.

**Appendix S3.** Natural selection on males.

**Appendix S4.** Effect of initial parameter values.

**Appendix S5.** Parameter combinations.

**Appendix S6.** Line of equilibria and conditions for runaway selection.
