# Supplementary material for: The evolution of phenotypes and genetic parameters under preferential mating
Source: Ecol Evol. 2014 Jun 11;4(13):2759–76. doi: 10.1002/ece3.1130 (PMC4113298; doi:10.1002/ece3.1130)
Supplement: Supplementary file 1 — Table S1. Details on the parameter combinations used. [file ece30004-2759-SD1.docx]

Supplementary Table S1. Details on the parameter combinations used.

| Parameter Levels | |
| --- | --- |
| Set 1 Data: AP model without natural selection | |
| $h_{xr}^{2}$ | 0.2 |
| $h_{yr}^{2}$ | 0.4 |
| *G*_ratio_ | 0.10, 0.20, 0.25, 0.40, 0.50, 0.80, 1.00, 1.60, 2.00, 3.00, 4.00 |
| $\nu$ | 10, 20, 30, 40, 50, 60 |
| *N* | 5, 20, 100 |
| We used all 198 possible combinations. | |
| Set 1 Data: AP model with natural selection and RP model with and without natural selection | |
| $h_{xr}^{2}$ | 0.2 |
| $h_{yr}^{2}$ | 0.4 |
| *G*_ratio_ | 0.25, 0.50, 1.00, 2.00, 4.00 |
| $\nu$ | 10, 20, 30, 40, 50, 60 |
| *N* | 5, 20, 100 |
| We used all 90 possible combinations. | |
| Set 2 Data | |
| $h_{xr}^{2}$ | 0.05, 0.10, 0.20, 0.40, 0.60 |
| $h_{yr}^{2}$ | 0.1, 0.2, 0.4, 0.5, 0.6, 0.8 |
| $\nu$ | 10, 20, 40 |
| *G*_ratio_ | 0.025, 0.05, 0.10, 0.20, 0.25, 0.40, 0.50, 1.00, 2.00, 4.00 |
| *N* | 5, 20, 100 |
| We used 901 of the 2700 possible combinations. | |
| Data set used to compare modes of choice | |
| $h_{xr}^{2}$ | 0.05, 0.10, 0.20, 0.40 |
| $h_{yr}^{2}$ | 0.1, 0.2, 0.4, 0.8 |
| $\nu$ | 10, 20, 40 |
| *G*_ratio_ | 0.25, 0.50, 1.00, 2.000 |
| *N* | 5, 20, 100 |
| We used all 506 possible combinations. | |
