# Supplementary material for: The evolution of phenotypes and genetic parameters under preferential mating
Source: Ecol Evol. 2014 Jun 11;4(13):2759–76. doi: 10.1002/ece3.1130 (PMC4113298; doi:10.1002/ece3.1130)
Supplement: Supplementary file 2 — Appendix S1. Mutation rate. [file ece30004-2759-SD2.docx]

**Appendix S1: Mutation rate**

The per locus mutation rate, *μ*, was determined assuming a mutational variance, *V*_m_, of 10^-5^ and the relationship $V_{m}=2n\mu/V_{e}$, where *n* is the number of loci (Reeve 2000), which upon rearrangement gives$\mu=V_{m}V_{e}/2n$. The number of mutations for each trait was then drawn from a Poisson distribution with mean $\lambda=2n\mu N_{pop}$where *N*_pop_ is population size (Reeve 2000).
