# Supplementary material for: The evolution of phenotypes and genetic parameters under preferential mating
Source: Ecol Evol. 2014 Jun 11;4(13):2759–76. doi: 10.1002/ece3.1130 (PMC4113298; doi:10.1002/ece3.1130)
Supplement: Supplementary file 3 — Appendix S2. Population size and drift. [file ece30004-2759-SD3.docx]

**Appendix S2: Population size and drift**

Simulations run for 10,000 generations showed that under random mating the heritabilities of preference and the preferred trait declined by an average of only 9.5% without natural selection applied to the males and 11.7% with natural selection applied to the males. Given the relatively minor effect of drift on the heritabilities, we have ignored its effect in our analyses.
