# Supplementary material for: The evolution of phenotypes and genetic parameters under preferential mating
Source: Ecol Evol. 2014 Jun 11;4(13):2759–76. doi: 10.1002/ece3.1130 (PMC4113298; doi:10.1002/ece3.1130)
Supplement: Supplementary file 4 — Appendix S3. Natural selection on males. [file ece30004-2759-SD4.docx]

**Appendix S3: Natural selection on males**

As in Lande’s model, stabilizing natural selection on males is determined by the Gaussian function

$$w(y)=e^{-\frac{1}{2}\left(\frac{y-\theta}{\omega}\right)^{2}}$$

where$w(y)$is the fitness of a male with trait value$y$, $\theta$ is the optimum trait value and $\omega$ is a measure of the strength of stabilizing selection. As is typical for analytical models in quantitative genetics, Lande assumed that natural selection on males is sufficiently weak that the variance lost due to selection is replaced by recurrent mutation. However, a value for “weak selection” is not specified. We consider two cases: first the limiting case of weak selection, namely no natural selection on males (i.e. $\omega$=∞, so that $w(y)=1$ for all values of *y*); and second, relatively strong natural selection ($\omega$=50 giving a mortality of males that typically exceeded 10% per generation).
