# Supplementary material for: The evolution of phenotypes and genetic parameters under preferential mating
Source: Ecol Evol. 2014 Jun 11;4(13):2759–76. doi: 10.1002/ece3.1130 (PMC4113298; doi:10.1002/ece3.1130)
Supplement: Supplementary file 5 — Appendix S4. Effect of initial parameter values. [file ece30004-2759-SD5.docx]

**Appendix S4: Effect of initial parameter values**

According to Lande’s model the initial values of these parameters should have no effect on the genetic architecture at equilibrium. To verify this prediction, we ran a series of simulations of the AP model with stabilizing natural selection on males to determine if displacing the natural selection optimum for the preferred trait from the initial mean female preference would alter the genetic correlation at equilibrium. In these runs, the initial mean female preference remained zero but *θ* was displaced from zero by as much as 100 units. As previously, the initial mean for the preferred trait was set equal to *θ.* A total of 330 different combinations of additive genetic variance for female preference and male trait were run. We analyzed these simulation results using stepwise regression to predict the genetic correlation at equilibrium with *G_Ratio_*, *ν*, *θ* and their interactions as independent variables. The resultant model explained 88% of the variance and did not include *θ* or its interaction terms with other variables (*r*=0.94, *F*_3,326_=772, *P*<0.0001). Thus, in accordance with Lande’s prediction, initial disparity between the mean female preference and the natural selection optimum male trait value does not affect the equilibrium genetic correlation between the preferred trait and the preference.
