# Supplementary material for: The evolution of phenotypes and genetic parameters under preferential mating
Source: Ecol Evol. 2014 Jun 11;4(13):2759–76. doi: 10.1002/ece3.1130 (PMC4113298; doi:10.1002/ece3.1130)
Supplement: Supplementary file 6 — Appendix S5. Parameter combinations. [file ece30004-2759-SD6.docx]

**Appendix S5: Parameter combinations**

Preliminary results indicated that the genetic correlation, the phenotypic correlation and the heritability of preference achieved equilibrium within 50 generations but it could take several thousand generations for the heritability of the preferred trait to reach equilibrium. Therefore, for set 1 we ran the simulations for 10,000 generations and computed mean values over the last 500 generations. For the set 1 data the initial values of $h_{xr}^{2}$and $h_{yr}^{2}$ were fixed at 0.2 and 0.4, respectively, which are reasonable values based on empirical estimates (Bakker & Pomiankowski 1995; Pomiankowski & Moller 1995; Prokuda & Roff unpubl. data). For the AP model without natural selection we used a total of 198 different combinations of parameter values in which the *G*_ratio_ was varied from 0.1 to 4, *υ* from 10 to 60 and *N* set equal to 5, 20 or 100 ( Table S5.1 ). We used a smaller data set consisting of 90 combinations for the analysis of the AP model with natural selection and the RP model with and without selection ( Table S5.1).

To determine if the values of the initial heritabilities influence the genetic correlation at equilibrium, we ran a second set of simulations (set 2) for the AP model without stabilizing natural selection and with the SE/BM mode of mate choice. Because the genetic correlation reaches equilibrium within 50 generations, we ran these simulations for only 100 generations and computed the genetic correlation over the last 20 generations. For this set we generated 901 combinations which varied $h_{xr}^{2}$ from 0.05 to 0.4, $h_{yr}^{2}$ from 0.1 to 0.8, *G*_ratio_ from 0.025 to 4, and *v* from 10 to 40, with *N* of 5, 20 or 100 ( Table S5.1). To examine the influence of the mode of female choice, we also ran a subset of 576 different combinations from set 2 with each of the three modes of choice (SE/BM, SI/BM and SE/LM: Table S5.1).

Appendix Table S5.1. Details on the parameter combinations used.

| Parameter Levels | |
| --- | --- |
| Set 1 Data: AP model without natural selection | |
| $h_{xr}^{2}$ | 0.2 |
| $h_{yr}^{2}$ | 0.4 |
| *G*_ratio_ | 0.10, 0.20, 0.25, 0.40, 0.50, 0.80, 1.00, 1.60, 2.00, 3.00, 4.00 |
| $\nu$ | 10, 20, 30, 40, 50, 60 |
| *N* | 5, 20, 100 |
| We used all 198 possible combinations. | |
| Set 1 Data: AP model with natural selection and RP model with and without natural selection | |
| $h_{xr}^{2}$ | 0.2 |
| $h_{yr}^{2}$ | 0.4 |
| *G*_ratio_ | 0.25, 0.50, 1.00, 2.00, 4.00 |
| $\nu$ | 10, 20, 30, 40, 50, 60 |
| *N* | 5, 20, 100 |
| We used all 90 possible combinations. | |
| Set 2 Data | |
| $h_{xr}^{2}$ | 0.05, 0.10, 0.20, 0.40, 0.60 |
| $h_{yr}^{2}$ | 0.1, 0.2, 0.4, 0.5, 0.6, 0.8 |
| $\nu$ | 10, 20, 40 |
| *G*_ratio_ | 0.025, 0.05, 0.10, 0.20, 0.25, 0.40, 0.50, 1.00, 2.00, 4.00 |
| *N* | 5, 20, 100 |
| We used 901 of the 2700 possible combinations. | |
| Data set used to compare modes of choice | |
| $h_{xr}^{2}$ | 0.05, 0.10, 0.20, 0.40 |
| $h_{yr}^{2}$ | 0.1, 0.2, 0.4, 0.8 |
| $\nu$ | 10, 20, 40 |
| *G*_ratio_ | 0.25, 0.50, 1.00, 2.000 |
| *N* | 5, 20, 100 |
| We used all 506 possible combinations. | |
