# Supplementary material for: The evolution of phenotypes and genetic parameters under preferential mating
Source: Ecol Evol. 2014 Jun 11;4(13):2759–76. doi: 10.1002/ece3.1130 (PMC4113298; doi:10.1002/ece3.1130)
Supplement: Supplementary file 7 — Appendix S6. Line of equilibria and conditions for runaway selection. [file ece30004-2759-SD7.docx]

**Appendix S6: Line of equilibria and conditions for runaway selection**

The line of equilibria between the preference and preferred trait predicted by Lande’s model is given by,

$$\begin{matrix} \text{Absolute preference model: }\bar{x}=\left(\frac{\nu^{2}}{\omega^{2}}+1\right)\bar{y}-\frac{\nu^{2}}{\omega^{2}}\theta\\ \text{Relative preference model: }\bar{x}=\left(\frac{\nu^{2}}{\omega^{2}}\right)\bar{y}-\frac{\nu^{2}}{\omega^{2}}\theta\end{matrix}$$

From Lande’s analysis we can derive the condition for runaway selection to occur as being,

$$\begin{matrix} \text{Absolute preference model: }r_{g}>\left(\frac{\nu^{2}}{\omega^{2}}+1\right)\sqrt{\frac{V_{Gy}}{V_{Gx}}} \\ \text{Relative preference model: }r_{g}>\left(\frac{\nu^{2}}{\omega^{2}}\right)\sqrt{\frac{V_{Gy}}{V_{Gx}}} \end{matrix}$$

where *V*_Gx_, *V*_Gy_ are the additive genetic variances in the preference and preferred trait, respectively. Note that in the absence of natural selection on the males (i.e. $\omega$ extremely large), the relative preference model will always cause runaway selection because the right hand side of the equation approaches zero.
